# Generation of PLASE score for patent ductus arteriosus using the PLASE study database

**DOI:** 10.1038/s41390-025-03803-w

**Published:** 2025-02-08

**Authors:** Satoshi Masutani, Tetsuya Isayama, Tohru Kobayashi, Kyongsun Pak, Seiichi Tomotaki, Hiroko Iwami, Takehiko Yokoyama, Katsuaki Toyoshima, On behalf of the PLASE (Patent ductus arteriosus and Left Atrial Size Evaluation in preterm infants) study group investigators, Satoshi Masutani, Tetsuya Isayama, Tohru Kobayashi, Hiroko Iwami, Takehiko Yokoyama, Katsuaki Toyoshima, Hiroyuki Nagasawa, Hidenori Kawasaki, Satoshi Masutani, Takehiko Yokoyama, Tohru Kobayashi, Chemin Su, Tetsuya Isayama, Eisuke Inoue, Masashi Mikami, Takehiko Yokoyama, Hidenori Kawasaki, Shun Matsumura, Atsushi Nakayama, Takashi Tachibana, Katsuaki Toyoshima, Toshifumi Ikeda, Naoko Okada, Kaoru Okazaki, Yutaka Yamamoto, Akane Honda, Atsushi Matsumoto, Shoichiro Amari, Kenichi Masumoto, Satoshi Onishi, Hiroko Iwami, Miki Yamaura, Masahiko Sato, Jun Shiraishi, Tomoyuki Kamamoto, Yusei Nakata, Mitsumaro Nii, Aiko Aoyama, Masahito Yamamoto, Hiroyuki Kitano, Hiroshi Sugiura, Takahide Yanagi, Tomoko Saito, Satoru Iwashima, Takamichi Ishikawa, Tomohiro Hayashi, Hiroki Ishii, Yusuke Suganami, Kenichi Tanaka, Atsushi Nakao, Masaki Kobayashi, Masafumi Miyata, Yoshiaki Sato, Nanae Yutaka, Atsushi Ohashi, Tomoko Fuke, Masayuki Miwa, Takenori Kato, Shin Kato

**Affiliations:** 1 Pediatrics, Saitama Medical Center, Saitama Medical University, Kawagoe, Japan; 2 Neonatology, National Center for Child Health and Development, Setagaya, Japan; 3 Department of Data Science, Clinical Research Center, National Center for Child Health and Development, Setagaya, Japan; 4 Department of Neonatology, Graduate School of Medicine, Kyoto University, Kyoto, Japan; 5 Department of Neonatology, Osaka City General Hospital, Osaka, Japan; 6 Department of Pediatrics, Japanese Red Cross Nagoya Daini Hospital, Nagoya, Japan; 7 Department of Neonatology, Kanagawa Children’s Medical Center, Yokohama, Japan; 8 Gifu Prefectural General Medical Center, Gifu, Japan; 9 Japanese Red Cross Nagoya Daiichi Hospital, Nagoya, Japan; 10 Nagoya University, Nagoya, Japan; 11 Aomori Prefectural Central Hospital, Aomori, Japan; 12 Tokyo Metropolitan Children’s Medical Center, Fuchu, Japan; 13 Hiroshima City Hiroshima Citizens Hospital, Hiroshima, Japan; 14 Iwate Medical University, Morioka, Japan; 15 Tokyo Women’s Medical University, Shinjuku, Japan; 16 Osaka City General Hospital, Osaka, Japan; 17 Tokyo Women’s Medical University Yachiyo Medical Center, Yachiyo, Japan; 18 Osaka Women’s and Children’s Hospital, Osaka, Japan; 19 Nara Medical University, Nara, Japan; 20 Kochi Health Sciences Center, Kochi, Japan; 21 Asahikawa-Kosei Hospital, Asahikawa, Japan; 22 Nagahama Red Cross Hospital, Nagahama, Japan; 23 Ishikawa Prefectural Central Hospital, Kanazawa, Japan; 24 Seirei Hamamatsu General Hospital, Hamamatsu, Japan; 25 Shiga University of Medical Science, Otsu, Japan; 26 Niigata University, Niigata, Japan; 27 Hamamatsu University School of Medicine, Hamamatsu, Japan; 28 Kurashiki Central Hospital, Kurashiki, Japan; 29 Tokyo Medical University, Shinjuku, Japan; 30 Kumamoto University, Kumamoto, Japan; 31 Japanese Red Cross Medical Center, Shibuya, Japan; 32 Sapporo Medical University, Sapporo, Japan; 33 Fujita Health University, Toyoake, Japan; 34 Yodogawa Christian Hospital, Osaka, Japan; 35 Kansai Medical University Hospital, Hirakata, Japan; 36 Saitama City Hospital, Saitama, Japan; 37 Nagoya City University, Nagoya, Japan

## Abstract

**Background:**

No echocardiographic model, to the best of our knowledge, has been established to predict the future need for patent ductus arteriosus (PDA) surgery. This study aimed to develop a novel predictive score (PLASE score) for anticipating the need for PDA surgery using the PLASE study database.

**Methods:**

The included infants with gestational age (GA) < 30 weeks were allocated to derivation and validation groups (2:1). Logistic regression models were constructed to predict the future need for PDA surgery utilizing three clinical and three echocardiographic indices measured at 3 days of age as candidate variables. ROC-AUCs and 95% confidence intervals (CIs) were obtained by 3-fold cross-validation and the percentile method, respectively. The model with the largest ROC-AUC was tested in the validation data.

**Results:**

Derivation and validation data included 463 and 229 patients, respectively, with 55 and 22 surgical cases, respectively. The ROC-AUC was maximized in the model using GA and all three echocardiographic indices (0.846 [95% CI, 0.805–0.886]). In the validation data, the ROC-AUC for the same model was 0.827 (0.744–0.911).

**Conclusions:**

We created a surgical prediction model using simple indices at 3 days of age, and the validation data demonstrated good predictive ability.

**Impact:**

- No early predictive model has been established for the future need of patent ductus arteriosus (PDA) surgery in preterm infants.
- A new prediction model was created with the Patent ductus arteriosus and Left Atrial Size Evaluation study in preterm infants (PLASE) database (*N* = 692), incorporating gestational age and three simple echocardiographic indices measured at 3 days of age. The model demonstrates high discrimination and calibration.
- This model provides risk stratification for preterm PDA and may contribute to early preterm management.

## Introduction

Patent ductus arteriosus (PDA)^1^ is a major complication in preterm infants, impacting neonatal outcomes. Surgery is indicated for severe PDA that are refractory to or contraindicated by pharmacotherapy. Transfer may be necessary to perform PDA surgery in some neonatal intensive care units (NICUs). An accurate prediction of the probability of the future need for PDA surgery would be clinically valuable. Although various scores have been proposed for PDA, to the best of our knowledge, no model has been established to accurately predict the future need for PDA surgery using both clinical and echocardiographic findings in a sufficiently large cohort. The Patent ductus arteriosus and Left Atrial Size Evaluation study in preterm infants (PLASE), conducted at 34 centers, demonstrated the usefulness of ductus arteriosus diameter (PDAd) and left pulmonary artery end-diastolic velocity (LPAedv) in comparing indices by evaluating the worst echocardiographic indices up to 2 weeks of age.^2^ In a subanalysis, echocardiographic data at 3 days of age also indicated that PDAd and LPAedv were predictive of future need for ductus arteriosus surgery.^3^ However, previous studies have been unable to quantitatively predict the future need for PDA surgery using a model integrating various clinical and echocardiographic data. Therefore, this study aimed to develop a quantitative model (PLASE score) for predicting the future need for PDA surgery based on clinical information and echocardiographic findings at 3 days of age using the PLASE study database.

## Methods

### Study design and patients

This study was a post-hoc analysis of the PLASE study, a prospective multicenter cohort study that included 710 preterm infants at 34 NICUs in Japan.^2^ The study^2^ had the following predefined inclusion criteria: (1) gestational age at birth between 23 weeks and 0 day and 29 weeks and 6 days, (2) admission to a participating NICU within 24 h of birth, and (3) surviving beyond the first 24 h after birth. The study^2^ excluded patients with (1) chromosomal abnormalities; (2) multiple anomalies, apparent clinical syndrome, or congenital anomalies that required surgery during infancy; (3) congenital heart disease other than PDA, patent foramen ovale, and persistent left superior vena cava; (4) congenital metabolic, endocrine, neuromuscular, or systemic bone disease; (5) family history of severe hereditary diseases such as neuromuscular disease or cardiomyopathy; and (6) critical conditions deemed ineligible for this study by attending physicians. Among them, we excluded patients who lacked any of the echocardiographic measurements of the PDAd, LPAedv, and left atrial to aortic diameter ratio (LA/Ao) at 3 days of age. We selected these three echocardiographic indices (PDAd, LPAedv, and LA/Ao) as potential dependent variables based on findings from a previous study,^3^ which identified them as having the three highest unadjusted and adjusted areas under the curve values for predicting future surgery. We also excluded patients who underwent PDA surgery before echocardiography evaluation at 3 days of age. The details of the echocardiographic examinations have been described in previous reports.^2,3^ All echocardiographers were assessed and met predefined quality control criteria for accurate echocardiographic measurements before data acquisition.^4^ Most were experienced neonatologists who routinely performed echocardiography to assess cardiac function and PDA among preterm infants in their daily clinical practice.^2,3^ The decision to surgically close the PDA was left to the discretion of the physicians or surgeons and was based on clinical conditions such as hemodynamic instability, feeding problems, and further renal or respiratory impairment.^2,3^

### Statistical analysis

Among the cases that did not meet the exclusion criteria, the data were divided for model derivation and validation. The initial two-thirds of cases from each center were used for model derivation, while the remaining one-third were reserved for validation. Descriptive statistics (e.g., mean ± standard deviation, median [minimum, maximum]) were used to summarize the demographic or clinical data of infants who did or did not undergo PDA surgery. We summarized the background data for the derivation and validation groups for patients with and without ductus arteriosus surgery to determine if the patients were appropriately subdivided. PDA surgery served as the independent variable, with three clinical variables (gestational age, small for gestational age (SGA)/no SGA, and birth body weight [BBW]) and three echocardiographic variables (PDAd, LPAedv, and LA/Ao) as predictors in the logistic regression model. When BBW was used, the model did not use gestational age and SGA/no SGA. Based on the number of PDA surgery cases in the derivation group, the upper limit of candidate variables included in the model was five. The following three modeling patterns were implemented.

- Candidate model scenario 1: a combination of a maximum of five variables (or four if BBW was included) without a mandatory variable.
- Candidate model scenario 2: a maximum of four variables using BBW as a mandatory variable.
- Candidate model scenario 3: a maximum of five variables using gestational age and SGA/no SGA as a mandatory variable.

The odds ratio and 95% CI were calculated for each candidate model, and the repeated 3-fold cross-validated ROC-AUC (200 times) and 95% CI, using the bootstrap method (1000 times), were calculated as an indicator of the prediction performance of the model. In addition, the Brier Score was calculated to evaluate prediction error.

From each candidate model scenario, the model with the maximum ROC-AUC was selected as the optimal score model. If multiple models had the same ROC-AUC value, the model with the least number of variables was selected based on the principle of parsimony.

Next, the optimal model for each selected candidate scenario was applied to the validation data, and the ROC-AUC, 95% CI with the same model as the derivation method, and Brier score were calculated. The calibration plot and intraclass correlation coefficient (ICC) were also calculated.

Finally, the best model for each selected candidate model pattern was re-estimated using all data integrated from the derivation and validation data, and the odds ratio, ROC-AUC, 95% CI, and Brier score were calculated. The predicted probability of PDA surgery by this logistic regression model was defined as the PLASE score. All analyses were conducted with R version 4.1.2.

## Results

### Demographics

**Fig. 1:**
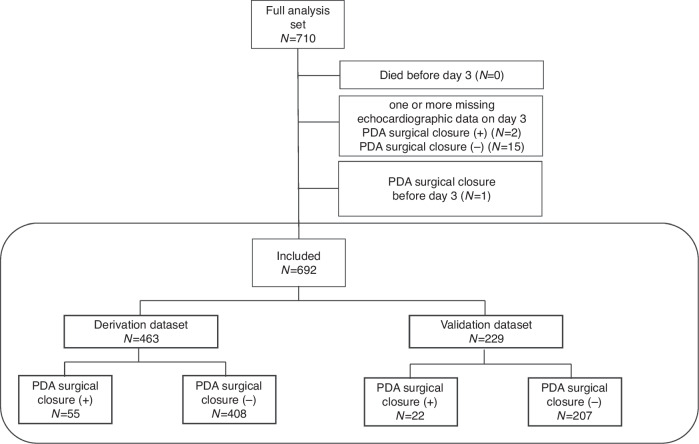
Flowchart of this study. Among the full analysis set of previous studies,^2,3^ we excluded 18 patients (2 with PDA surgical closure and 15 without PDA surgical closure) lacking any of the echocardiographic measurements of the PDA diameter, left pulmonary arterial end-diastolic velocity and left atrial to aortic diameter ratio at 3 days of age. We also excluded one patient who underwent PDA surgery before echocardiography evaluation at 3 days of age.^3^ Subsequently, cases that corresponded to the initial two-thirds of cases at each center were used as data for model derivation, and those that corresponded to the remaining one-third of cases were used as data for validation. PDA, patent ductus arteriosus.

As illustrated in Fig. 1, from the full analysis set in previous studies,^2,3^ we excluded 17 patients (2 with PDA surgical closure and 15 without PDA surgical closure) lacking any of the echocardiographic measurements of PDAd, LPAedv, and LA/Ao at 3 days of age. We also excluded one patient who underwent PDA surgery before evaluation of the echocardiography at 3 days of age. The details of the echocardiographic examinations have been described in previous reports.^2,3^ Finally, 692 patients (77 with PDA surgical closure and 615 without PDA surgical closure) were included in this study. Of the 77 patients who underwent PDA surgical closure, 33 (42.9%) and 71 (92.2%) received prophylactic indomethacin and a therapeutic cyclooxygenase inhibitor, respectively. Among the 615 patients without PDA surgical closure, 185 (30.1%) received prophylactic indomethacin and 300 (48.8%) received therapeutic cyclooxygenase inhibitors.

Table 1 shows the demographic data of cases that corresponded to the initial two-thirds of the cases (*N* = 463) at each center for model creation and those that corresponded to the remaining one-third of the cases (*N* = 229) for model validation. The background data for the derivation and validation groups for patients with and without PDA surgery indicated that the patients were appropriately subdivided.

**Table 1:** Characteristics of patients.

	Derivation dataset		Validation dataset	
	PDA surgical closure	No PDA surgical closure	PDA surgical closure	No PDA surgical closure
Demographics				
Number of patients	55	408	22	207
Gestational age (weeks)	25.2 ± 1.6	26.5 ± 1.8	24.7 ± 1.7	26.6 ± 1.8
Birth weight (g)	750 ± 192	883 ± 262	714 ± 125	870 ± 277
Birth height (cm)	31.5 ± 2.7	33.3 ± 3.5	31.0 ± 2.8	33.2 ± 3.5
Small for gestational age	10 (18.2%)	90 (22.1%)	2 (9.1%)	54 (26.1%)
Singleton (%)	36 (65.5%)	321 (78.7%)	20 (90.9%)	183 (88.4%)
Male sex (%)	27 (49.1%)	224 (54.9%)	8 (36.4%)	118 (57.0%)
In-hospital birth (%)	55 (100.0%)	402 (98.5%)	21 (95.5%)	202 (97.6%)
Cesarean section (%)	48 (87.3%)	335 (82.1%)	20 (90.9%)	172 (83.1%)
No antenatal steroid (%)	12 (21.8%)	105 (25.7%)	5 (22.7%)	45 (21.7%)
1 time (%)	8 (14.5%)	47 (11.5%)	5 (22.7%)	26 (12.6%)
2 times (%)	34 (61.8%)	256 (62.7%)	12 (54.5%)	136 (65.7%)
Apgar score, 1 min	3.8 ± 2.1	4.1 ± 2.2	3.4 ± 2.2	4.4 ± 2.0
Apgar score, 5 min	6.2 ± 1.9	6.4 ± 2.1	6.3 ± 1.5	7.0 ± 1.7
Prophylactic indomethacin (%)	23 (41.8%)	128 (31.4%)	10 (45.5%)	57 (27.5%)
Therapeutic cyclooxygenase inhibitor (%)	51 (92.7%)	199 (48.8%)	20 (90.9%)	101 (48.8%)
Echocardiographic indices at day 3				
LAV (mL)	0.536 ± 0.376	0.490 ± 0.278	0.435 ± 0.154	0.493 ± 0.233
PDAd (mm)	1.01 ± 1.00	0.30 ± 0.54	0.79 ± 0.70	0.30 ± 0.55
LVDd (mm)	11.4 ± 2.1	11.8 ± 1.8	10.9 ± 2.0	11.8 ± 2.1
LPAedv (cm/s)	12.3 ± 9.8	5.2 ± 8.5	13.8 ± 14.2	4.7 ± 6.6
LA/Ao	1.35 ± 0.23	1.21 ± 0.19	1.22 ± 0.26	1.21 ± 0.21
LAV (mL)/BBW (kg)	0.692 ± 0.359	0.564 ± 0.277	0.612 ± 0.205	0.576 ± 0.231
PDAd (mm)/BBW (kg)	1.30 ± 1.23	0.34 ± 0.66	1.11 ± 1.08	0.36 ± 0.69
LVDd (mm)/BBW (kg)	15.8 ± 3.1	14.2 ± 3.3	15.4 ± 2.5	14.5 ± 3.7
Outcome related variable				
PDA surgery performed (day)	20 ± 11	–	21 ± 10	–

*LAV* left atrial volume, *PDAd* patent ductus arteriosus diameter, *LVDd* left ventricular diastolic dimension, *LPAedv* left pulmonary artery end-diastolic velocity, *LA/Ao* left atrial to aortic diameter ratio, *BBW* birth body weight.

The predictive abilities for future need for PDA surgery of candidate model scenario 1 (39 models), candidate model scenario 2 (8 models), and candidate model scenario 3 (8 models) are summarized in Table 2. Among those, model 33, which uses four indices (PDAd, LPAedv, LA/Ao, and gestational age), was selected as the best model in candidate model scenario 1, with the highest ROC-AUC (0.845 [95% CI, 0.796–0.894]). This model was one of several with the lowest Brier scores (0.081), indicating being a good model in light of prediction errors. Model 47, which uses four indices (PDAd, LPAedv, LA/Ao, and BBW), was selected as the best model in candidate model scenario 2, with an ROC-AUC of 0.815 (95% CI, 0.754–0.875) and a Brier score of 0.083. Model 55, which uses five indices (PDAd, LPAedv, LA/Ao, gestational age, and SGA/no SGA), was selected as the best model in candidate model scenario 3, with an ROC-AUC of 0.842 (95% CI, 0.791–0.892) and a Brier score of 0.081.

**Table 2:** ROC-AUCs with derivation dataset.

	PDAd OR (95%CI)	LPAedv OR (95%CI)	LA/Ao OR (95%CI)	Gestational age (weeks) OR (95%CI)	SGA OR (95%CI)	BBW OR (95%CI)	ROC-AUC (95%CI)	Brier Score
Candidate Model Scenario 1								
Model1	3.55 (2.40–5.24)	–	–	–	–	–	0.728 (0.657–0.799)	0.091
Model2	–	1.07 (1.04–1.09)	–	–	–	–	0.737 (0.667–0.807)	0.1
Model3	–	–	23.64 (6.42–87.01)	–	–	–	0.693 (0.622–0.764)	0.099
Model4	–	–	–	0.67 (0.56–0.79)	–	–	0.703 (0.634–0.771)	0.099
Model5	–	–	–	–	0.79 (0.38–1.62)	–	0.508 (0.443–0.572)	0.105
Model6	–	–	–	–	–	1.00 (1.00–1.00)	0.649 (0.578–0.720)	0.102
Model7	2.98 (1.89–4.72)	1.02 (0.99–1.05)	–	–	–	–	0.741 (0.666–0.817)	0.09
Model8	3.01 (1.99–4.53)	–	7.94 (1.90–33.12)	–	–	–	0.757 (0.687–0.828)	0.089
Model9	4.73 (3.02–7.39)	–	–	0.57 (0.47–0.70)	–	–	0.835 (0.787–0.884)	0.084
Model10	3.54 (2.39–5.23)	–	–	–	0.93 (0.42–2.03)	–	0.726 (0.653–0.799)	0.091
Model11	–	1.05 (1.03–1.08)	15.97 (4.08–62.55)	–	–	–	0.760 (0.692–0.829)	0.096
Model12	–	1.08 (1.05–1.11)	–	0.63 (0.52–0.75)	–	–	0.805 (0.751–0.858)	0.094
Model13	–	1.07 (1.04–1.09)	–	–	0.73 (0.33–1.58)	–	0.725 (0.652–0.798)	0.1
Model14	–	–	20.36 (5.11–81.12)	0.67 (0.57–0.80)	–	–	0.771 (0.708–0.834)	0.095
Model15	–	–	25.64 (6.87–95.66)	–	0.67 (0.31–1.43)	–	0.690 (0.618–0.762)	0.099
Model16	–	–	–	0.66 (0.56–0.79)	1.15 (0.54–2.46)	–	0.698 (0.625–0.770)	0.099
Model17	4.67 (2.99–7.27)	–	–	–	–	1.00 (0.99–1.00)	0.805 (0.743–0.868)	0.084
Model18	–	1.07 (1.04–1.10)	–	–	–	1.00 (1.00–1.00)	0.784 (0.723–0.844)	0.097
Model19	–	–	19.23 (5.04–73.45)	–	–	1.00 (1.00–1.00)	0.733 (0.665–0.800)	0.097
Model20	2.56 (1.57–4.17)	1.02 (0.99–1.05)	7.68 (1.81–32.55)	–	–	–	0.761 (0.690–0.832)	0.089
Model21	3.96 (2.32–6.74)	1.02 (0.98–1.06)	–	0.57 (0.47–0.70)	–	–	0.839 (0.791–0.886)	0.084
Model22	2.96 (1.87–4.69)	1.02 (0.99–1.05)	–	–	0.88 (0.40–1.95)	–	0.738 (0.663–0.814)	0.09
Model23	4.08 (2.56–6.51)	–	5.30 (1.15–24.37)	0.58 (0.47–0.71)	–	–	0.842 (0.794–0.891)	0.082
Model24	2.97 (1.97–4.49)	–	8.31 (1.97–35.07)	–	0.81 (0.36–1.82)	–	0.756 (0.686–0.825)	0.089
Model25	4.87 (3.09–7.67)	–	–	0.56 (0.45–0.69)	1.66 (0.70–3.92)	–	0.834 (0.783–0.885)	0.083
Model26	–	1.07 (1.04–1.10)	14.03 (3.26–60.45)	0.63 (0.52–0.76)	–	–	0.826 (0.774–0.879)	0.09
Model27	–	1.06 (1.03–1.08)	17.11 (4.34–67.46)	–	0.64 (0.29–1.42)	–	0.757 (0.689–0.825)	0.095
Model28	–	1.08 (1.04–1.11)	–	0.62 (0.51–0.75)	1.15 (0.51–2.60)	–	0.800 (0.744–0.856)	0.094
Model29	–	–	20.46 (5.10–82.14)	0.68 (0.56–0.81)	0.97 (0.44–2.17)	–	0.765 (0.701–0.830)	0.095
Model30	4.04 (2.40–6.82)	1.02 (0.98–1.05)	–	–	–	1.00 (0.99–1.00)	0.808 (0.748–0.869)	0.084
Model31	4.06 (2.55–6.46)	–	4.66 (1.01–21.56)	–	–	1.00 (1.00–1.00)	0.812 (0.750–0.874)	0.083
Model32	–	1.06 (1.03–1.09)	13.26 (3.22–54.63)	–	–	1.00 (1.00–1.00)	0.801 (0.740–0.862)	0.093
**Model33**	**3.40 (1.94–5.94)**	**1.02 (0.98–1.06)**	**5.34 (1.15–24.87)**	**0.58 (0.47–0.71)**	**–**	**–**	**0.845 (0.796–0.894)**	**0.081**
Model34	2.51 (1.53–4.11)	1.02 (0.99–1.06)	8.07 (1.88–34.52)	–	0.78 (0.35–1.77)	–	0.760 (0.690–0.830)	0.089
Model35	4.11 (2.40–7.04)	1.02 (0.98–1.06)	–	0.56 (0.45–0.69)	1.63 (0.69–3.86)	–	0.836 (0.786–0.887)	0.083
Model36	4.21 (2.61–6.77)	–	4.94 (1.05–23.17)	0.56 (0.45–0.70)	1.50 (0.62–3.63)	–	0.839 (0.788–0.889)	0.081
Model37	–	1.07 (1.04–1.10)	13.99 (3.24–60.40)	0.63 (0.52–0.77)	1.04 (0.45–2.45)	–	0.822 (0.768–0.876)	0.09
Model38	3.50 (2.02–6.07)	1.02 (0.98–1.06)	4.68 (1.00–21.85)	–	–	1.00 (1.00–1.00)	0.815 (0.754–0.875)	0.083
Model39	3.51 (2.00–6.17)	1.02 (0.98–1.06)	5.04 (1.06–23.90)	0.56 (0.45–0.70)	1.49 (0.61–3.61)	–	0.842 (0.791–0.892)	0.081
Candidate Model Scenario 2								
Model40	–	–	–	–	–	1.00 (1.00–1.00)	0.649 (0.578–0.720)	0.102
Model41	4.67 (2.99–7.27)	–	–	–	–	1.00 (0.99–1.00)	0.805 (0.743–0.868)	0.084
Model42	–	1.07 (1.04–1.10)	–	–	–	1.00 (1.00–1.00)	0.784 (0.723–0.844)	0.097
Model43	–	–	19.23 (5.04–73.45)	–	–	1.00 (1.00–1.00)	0.733 (0.665–0.800)	0.097
Model44	4.04 (2.40–6.82)	1.02 (0.98–1.05)	–	–	–	1.00 (0.99–1.00)	0.808 (0.748–0.869)	0.084
Model45	4.06 (2.55–6.46)	–	4.66 (1.01–21.56)	–	–	1.00 (1.00–1.00)	0.812 (0.750–0.874)	0.083
Model46	–	1.06 (1.03–1.09)	13.26 (3.22–54.63)	–	–	1.00 (1.00–1.00)	0.801 (0.740–0.862)	0.093
**Model47**	**3.50 (2.02–6.07)**	**1.02 (0.98–1.06)**	**4.68 (1.00–21.85)**	**–**	**–**	**1.00 (1.00–1.00)**	**0.815 (0.754–0.875)**	**0.083**
Candidate Model Scenario 3								
Model48	–	–	–	0.66 (0.56–0.79)	1.15 (0.54–2.46)	–	0.698 (0.625–0.770)	0.099
Model49	4.87 (3.09–7.67)	–	–	0.56 (0.45–0.69)	1.66 (0.70–3.92)	–	0.834 (0.783–0.885)	0.083
Model50	–	1.08 (1.04–1.11)	–	0.62 (0.51–0.75)	1.15 (0.51–2.60)	–	0.800 (0.744–0.856)	0.094
Model51	–	–	20.46 (5.10–82.14)	0.68 (0.56–0.81)	0.97 (0.44–2.17)	–	0.765 (0.701–0.830)	0.095
Model52	4.11 (2.40–7.04)	1.02 (0.98–1.06)	–	0.56 (0.45–0.69)	1.63 (0.69–3.86)	–	0.836 (0.786–0.887)	0.083
Model53	4.21 (2.61–6.77)	–	4.94 (1.05–23.17)	0.56 (0.45–0.70)	1.50 (0.62–3.63)	–	0.839 (0.788–0.889)	0.081
Model54	–	1.07 (1.04–1.10)	13.99 (3.24–60.40)	0.63 (0.52–0.77)	1.04 (0.45–2.45)	–	0.822 (0.768–0.876)	0.09
**Model55**	**3.51 (2.00–6.17)**	**1.02 (0.98–1.06)**	**5.04 (1.06–23.90)**	**0.56 (0.45–0.70)**	**1.49 (0.61–3.61)**	**–**	**0.842 (0.791–0.892)**	**0.081**

The model selected for each scenario is highlighted in yellow.

*PDAd* patent ductus arteriosus diameter, *LPAedv* left pulmonary artery end-diastolic velocity, *LA/Ao* left atrial to aortic diameter ratio, *SGA* small for gestational age, *BBW* birth body weight. Each model shown in bold is the best model in candidate model scenarios 1–3.

**Fig. 2:**
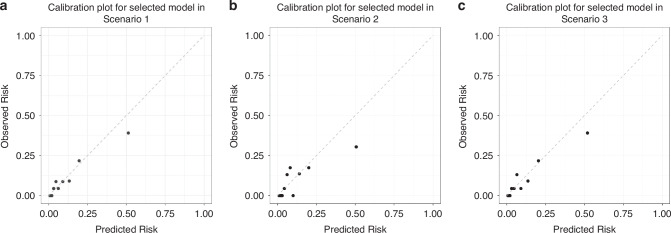
Calibration plot. Calibration plots of scenario 1(**a**), 2(**b**), and 3(**c**) were displayed. All were close to a straight line with y = x, and the best fit was seen in scenario 1.

As presented in Table 3, the optimal model for each selected candidate model scenario using the data for model derivation was validated using the data for validation. Among the three scenarios, model 33 in scenario 1 was selected as the best with an ROC-AUC of 0.827 (95% CI, 0.744–0.911), a Brier score of 0.075, and an ICC of 0.949 (95% CI, 0.821–0.987). As shown in Fig. 2, calibration plots in model 33 in scenario 1 (as well as model 55 in scenario 3) indicated satisfactory fitting in that a close match existed between the plots and the dashed ideal line, indicating a strong consistency between the observed probability and predicted probability of future need for PDA surgery.

**Table 3:** ROC-AUCs and ICCs in validation dataset.

	Model	ROC-AUC (95%CI)	Brier Score	ICC (95%CI), *p*-value
Selected Model in Scenario 1	PDAd + LPAedv + LA/Ao + GA	0.827 (0.744–0.911)	0.075	0.949 (0.821–0.987), < 0.001
Selected Model in Scenario 2	PDAd + LPAedv + LA/Ao + BBW	0.766 (0.674–0.858)	0.084	0.797 (0.403–0.945), 0.001
Selected Model in Scenario 3	PDAd + LPAedv + LA/Ao + GA + SGA	0.823 (0.738–0.907)	0.076	0.935 (0.776–0.983), < 0.001

*PDAd* patent ductus arteriosus diameter, *LPAedv* left pulmonary artery end-diastolic velocity, *LA/Ao* left atrial to aortic diameter ratio, *GA* gestational age, *BBW* birth body weight, *SGA* small for gestational age.

Finally, the best model for each selected candidate model pattern was re-estimated using all data integrated from the derivation and validation data, and the odds ratio, 95% CI, 200 times repeated 3-fold ROC-AUC, and Brier score were calculated (Table 4). Model 33 in scenario 1 was selected as the best, with an ROC-AUC of 0.846 (95% CI, 0.805–0.886) and a Brier score of 0.1. Finally, the proposed model in this study (PLASE score) is as follows:

$${{\rm{P}}}=	 \,1/\left(\right.1+\exp \left[-\left(\left(10.7091\right)+\left(1.141\right)* {{\rm{PDAd}}}+\left(0.036\right)* {{\rm{LPAedv}}} \right.\right.\\ 	\left.\left.+\left(0.6356\right)* {{\rm{LA}}}/{{\rm{A}}}0+\left(-0.5652\right)* {{\rm{GA}}}\right)\right]$$

**Table 4:** ORs and ROC-AUCs with entire dataset and score equations.

	PDAd OR (95%CI), *p*-value	LPAedv OR (95%CI), *p*-value	LA/Ao OR (95%CI), *p*-value	Gestational age (weeks) OR (95%CI), *p*-value	SGA OR (95%CI), *p*-value	BBW OR (95%CI), *p*-value	ROC-AUC (95%CI)	Brier Score
Selected Model in Scenario 1	3.13 (1.98–4.95), <0.001	1.04 (1.01–1.07), 0.020	1.89 (0.53–6.74), 0.327	0.57 (0.48–0.67), <0.001	–	–	0.846 (0.805–0.886)	0.1
Selected Model in Scenario 2	3.04 (1.97–4.69), < 0.001	1.03 (1.01–1.06), 0.019	1.61 (0.47–5.53), 0.447	–	–	1.00 (1.00–1.00), <0.001	0.807 (0.758–0.856)	0.1
Selected Model in Scenario 3	3.14 (1.98–4.98), < 0.001	1.04 (1.01–1.07), 0.020	1.87 (0.52–6.73), 0.335	0.57 (0.48–0.67), < 0.001	1.05 (0.50–2.21), 0.904	–	0.843 (0.802–0.884)	0.1
Model	Probability Equation							
Scenario 1	= 1/(1 + exp(−((10.7091) + (1.141)*PDAd + (0.036)*LPAedv + (0.6356)*LA/Ao + (−0.5652)*Gestational age)))							
Scenario 2	= 1/(1 + exp(−((−0.9883) + (1.1111)*PDAd + (0.0329)*LPAedv + (0.4784)*LA/Ao + (−0.0032)*BBW)))							
Scenario 3	= 1/(1 + exp(−((10.7766) + (1.1443)*PDAd + (0.0359)*LPAedv + (0.6282)*LA/Ao + (−0.5679)*Gestational age + (0.0459)*SGA (Yes = 1, No = 0))))							

*PDAd* patent ductus arteriosus diameter, *LPAedv* left pulmonary artery end-diastolic velocity, *LA/Ao* left atrial to aortic diameter ratio, *BBW* birth body weight, *SGA* small for gestational age.

## Discussion

This post-hoc study, utilizing the PLASE database with a sufficient sample size (*N* = 692) of early preterm infants in Japan, provided a prediction model for future need for PDA surgery using simple clinical and echocardiographic variables at 3 days of age, and cross-validation was performed. The model demonstrated high reliability, with ROC-AUC and ICC values of >0.8 and >0.9, respectively, and a low Brier score of 0.075 in the validation cohort. These results suggest that this model is currently the best available for predicting the future need for PDA surgery in early preterm infants at 3 days of age.

Previously PDA severity scores have been reported, but these models were based on limited sample sizes and lacked cross-validation. For instance, El-Khuffash et al. provided a PDA severity score using echocardiography data (PDAd, maximum PDA velocity, LV output, and late diastolic peak velocity of the lateral mitral annulus (a’) using tissue Doppler imaging) at 2 days of age.^5^ They reported the usefulness of the model, including the use of variables such as gestational age to predict chronic lung disease or death before discharge.^5^ Fink et al. and El-Khuffash et al. used the same cohort^5^ and compared the Shaare Zedek medical center (SZMC) score with the El-Khuffash model.^5,6^ The SZMC score uses the PDAd, LA/Ao, abdominal aorta, and PDA flow patterns,^6^ and is believed to be a hemodynamic index showing how PDA is symptomatic like our PDA PLASE score. The SZMC score was well correlated with the El-Khuffash score,^5,6^ and the El-Khuffash score^5^ appeared to have considerable overlap in the three groups classified by severity according to the SZMC score.^6^ El-Khuffash et al.^5^ and SZMC^6^ used the same cohort consisting of 141 preterm infants with a mean gestational age of 26.8 ± 1.4 weeks, including only 10 infants who underwent PDA surgery. McNamara and Sehgal model, which incorporates PDAd, transductal flow, LA/Ao, the ratio of peak mitral flow velocities during early and late diastole or isovolumetric relaxation time, and decreased or absent diastolic flow in superior mesenteric artery, middle cerebral artery, or renal artery^7^ provides information on PDA significance and has been used in multiple studies.^8^ However, we could not compare the PLASE score with the scores developed by El-Khuffash,^5^ SZMC,^6^ or McNamara and Sehgal^7^ because our model uses only three simple echocardiographic indices (PDAd, LPAedv, and LA/Ao). Our study did not capture tissue Doppler, LV output, transmitral flow, superior mesenteric/ middle cerebral/ renal arterial flow, or abdominal aortic flow which are necessary for calculating those other scores.^5–7^ To our knowledge, no previous studies including these three studies,^5–7^ have developed a PDA severity score using echocardiography based on a sufficiently large sample of infants and validated through appropriate cross-validation.

### Clinical implications

**Fig. 3:**
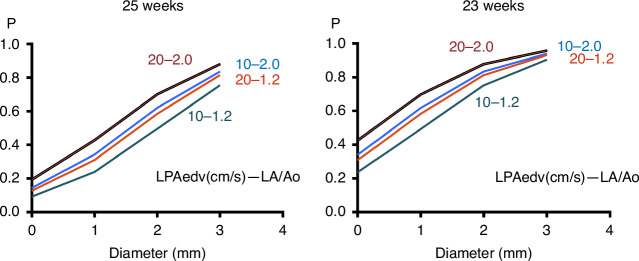
Prediction model. The relationship between the PLASE score (probability of future surgical PDA closure, vertical axis) and PDA diameter (horizontal axis) in the four representative settings with LA/Ao ratio of 1.2 and 2.0, and with LPAedv of 10 and 20 cm/s in infants with gestational ages of 23 and 25 weeks. PLASE, the Patent ductus arteriosus and Left Atrial Size Evaluation study in preterm infants; PDA, patent ductus arteriosus; LA/Ao, left atrial to aortic diameter; LPAedv, left pulmonary artery end-diastolic velocity.

The previous studies^2,3^ have not been able to quantitatively predict future need for PDA surgery by a model that integrates various clinical and echocardiographic information. This study advanced to provide a simple but reliable prediction model to predict future need for PDA surgery using GA and three easily measurable echocardiographic variables at 3 days of age with high discrimination and calibration ability. Such prediction of the future need of PDA surgical closure will help neonatologist’s decision-making process on the use of cyclooxygenase inhibitors, respiratory management, or blood transfusion.^3^ This model easily yields the probability using a spreadsheet. To help understand the nature of this prediction model, we determined the relationship between the PLASE score and PDAd in the four representative settings with LA/Ao ratio of 1.2 and 2.0, and with LPAedv of 10 and 20 cm/s in infants with gestational ages of 23 and 25 weeks (Fig. 3). The results indicated that the PDAd at 3 days of age plays a major role to predict future need for PDA surgery. This aligns with a previous report that gestational age and PDAd were the most important determinants of PDA treatment.^9^ Although PDAd may be expected to be the most important determinant to predict the need for PDA surgery in symptomatic infants with large PDAd, it is not the case. PDA surgical closure was performed at a mean age of 20–21 days in the included patients, and they were treated by various management strategies after 3 days of age. The PLASE score can reasonably provide information on the PDA status at 3 days of age with respect to the probability of future need for PDA surgical closure in the Japanese real-world practice and may contribute to optimize neonatal management.

### Study limitations

This study has a potential limitation inherent in the heterogeneity of the 34 institutes and multiple echocardiographers ( > 200).^2^ Nonetheless, the findings reflect the real-world practice in Japan. All echocardiographers, mostly neonatologists, had been evaluated and met the predefined quality control criteria for accurate echocardiographic measurements before data acquisition.^4^ Environmental differences for PDA surgical closure (five institutes [15%] needed to transfer patients, and six [18%] needed to invite surgeons for PDA surgical closure) may have affected the management and decision-making for the surgical closure of PDA.^2^ The indication criteria for PDA surgical closure were not standardized; instead, the decision for PDA surgical closure was clinically based on careful assessments of respiratory, renal, intestinal, and nutritional conditions as well as the overall tendency for the general condition to deteriorate.^2^ The current analysis set PDA surgical closure as the primary outcome and did not assess whether the PLASE score is useful for predicting death, severe bronchopulmonary dysplasia, or a composite of these two. These issues need to be investigated in future studies. Finally, international debates and differences in PDA therapies^10–12^ and surgical closure exist. Although the rate of PDA surgery of approximately 11% in the present cohort was comparable to that in other countries,^13^ the validity of the PLASE score outside Japan remains to be elucidated.

## Conclusions

We developed a predictive model for surgery using the echocardiographic index at 3 days of age from a large number of cases, and the validation data demonstrated robust predictive ability for surgery. In the future, we would like to examine the usefulness of the PLASE score as a predictive index for surgery and severity of disease in a new cohort of patients in a prospective study.

## Data Availability

The datasets generated during and/or analyzed during the current study are available from the corresponding author on reasonable request.
